# New Targeted Therapies for Thyroid Cancer

**DOI:** 10.2174/138920211798120808

**Published:** 2011-12

**Authors:** Alessandro Antonelli, Poupak Fallahi, Silvia M Ferrari, Ilaria Ruffilli, Francesca Santini, Michele Minuto, David Galleri, Paolo Miccoli

**Affiliations:** 1 Department of Internal Medicine, University of Pisa School of Medicine, Pisa, Italy; 2 Department of Surgery, University of Pisa School of Medicine, Pisa, Italy

**Keywords:** Anaplastic thyroid cancer, targeted molecular therapies, tyrosine kinase inhibitors, aurora kinase inhibitors, peroxisome proliferator-activated receptor-γ, RET, BRAF, VEGFR.

## Abstract

The increasing incidence of thyroid cancer is associated with a higher number of advanced disease characterized by the loss of cancer differentiation and metastatic spread. The knowledge of the molecular pathways involved in the pathogenesis of thyroid cancer has made possible the development of new therapeutic drugs able to blockade the oncogenic kinases (BRAF V600E, RET/PTC) or signaling kinases [vascular endothelial growth factor receptor (VEGFR), platelet-derived growth factor receptors (PDGFR)] involved in cellular growth and proliferation. Some clinical trials have been conducted showing the ability of targeted therapies (sorafenib, sunitinib, axitinib, imanitib, vandetanib, pazopanib, gefitinib) in stabilizing the course of the disease. Until now, however, no consensus guidelines have been established for patient selection and more data on toxicities and side effects are needed to be collected.

## INTRODUCTION

The incidence of thyroid carcinoma is rising faster than many other cancers, with the highest overall rate of increase in cancer deaths [1]. 

Differentiated thyroid cancer (DTC) is usually treated by total thyroidectomy followed by radioactive iodine (^131^I) ablation and thyroid hormone suppression of serum thyroid stimulating hormone (TSH). While cure is generally attainable in well-differentiated thyroid cancers [papillary (PTC) and follicular (FTC) types], recurrence occurs in up to 40% of patients [2]. In the course of the disease in about 5% of patients with thyroid cancer recurrence, the tumor becomes dedifferentiated (DeDTC) and unresponsive to ^131^I treatment [3], and it is usually accompanied by more aggressive growth and metastatic spread [4,5]. So the traditional therapeutic modalities of treatment lose their effectiveness. 

Radiotherapy and chemotherapy are the most popular treatment options for advanced stages of DTC and medullary thyroid carcinoma (MTC), but they confer only a modest benefit on tumor burden and overall survival. Their use is also limited by their toxicities. Current treatment regimens for advanced thyroid cancer include bleomycin, doxorubicin, platinum-containing compounds or a combination of these agents. 

Recent research in the field of the molecular mechanisms of thyroid cancer has underlined the role of oncogenic kinases, in particular in metastatic thyroid cancer [6]. Because of the high incidence of thyroid cancer, this field has become a focus of effort for use of new targeted therapies, especially the new class of agents that inhibit kinases involved in signaling, cellular growth, and angiogenesis [6]. Most of the therapeutic agents being developed actually target both the oncogenic and the signaling pathways.

## MEDULLARY THYROID CANCER AND *RET*

MTC is a rare tumor arising from neural crest-derived parafollicular C cells. Sporadic MTC is the most common form (70%). Hereditary MTC is associated with activating germline mutations of the *RET* proto-oncogene. Hereditary MTC manifests as part of the Multiple Endocrine Neoplasia Type 2 (MEN2) syndrome [7], transmitted as an autosomal dominant trait. 

The *RET* gene is located on chromosome 10q11.2 [8]. *RET* encodes a transmembrane receptor with an extracellular portion containing four calcium-dependent cell-adhesion domains that mediate the conformational properties needed to interact with ligands [9]. The extracellular portion of the receptor also contains multiple glycosylation sites and a cysteine-rich region necessary for the tertiary structure of the protein and for receptor dimerization [10]. The intracellular domain of the RET receptor contains two tyrosine kinase regions that activate intracellular signal transduction pathways. RET activation triggers autophosphorylation of tyrosine residues, that serve as docking sites for adaptor proteins, which coordinate cellular signal transduction pathways (e.g., mitogen-activated protein kinase, phosphatidylinositol 3-kinase, AKT, Jun N-terminal kinase, extracellular signal-regulated protein kinase) which are important in the regulation of cell growth [10]. The mutations of the *RET* gene in MTC lead to an activation of RET.

MEN2A is the most common (50%) form of hereditary MTC. There are three variants of MEN2A. The first is associated with Hirschsprung disease [11], the second is associated with cutaneous lichen amyloidosis [12] while the third variant, familial MTC (FMTC) [13] is characterized by MTC but no other manifestations of MEN2. MEN2A is characterized by bilateral, multicentric MTC in more than 90% of index patients (carrying germline *RET* mutations in codon 634; *i.e.* those presenting with MTC at diagnosis in the absence of screening), pheochromocytomas in 50% and primary hyperparathyroidism in 10 to 20%.

MEN2B is less common than MEN2A (approximately 20% of cases of MEN2) [14]. MEN2B is characterized by MTC in 100% of carriers, pheochromocytomas in 50%, mucosal ganglioneuromas in more than 90%, and a marphanoid habitus in nearly all. Early identification of MTC in MEN2B is important because metastases have been described during the first year of life.

The only possible curative treatment against MTC is its complete surgical resection, when possible [15]. Metastatic MTC patients are incurable because the cancer does not respond to radiotherapy or chemotherapy. The identification of activating mutations of the RET tyrosine kinase receptor in both hereditary and sporadic MTC makes this malignancy an excellent model to examine the effect of a group of small organic molecule tyrosine kinase inhibitors (TKIs) for the treatment of metastatic MTC.

## OVERVIEW ON MOLECULAR PATHWAYS INVOLVED IN THYROID CANCER DERIVED FROM FOLLICULAR CELLS

Of prime importance has been recognition of key oncogenic mutations in DTC and DeDTC. *BRAF* and *RAS* genes code for kinases that activate signaling through the mitogen activated protein kinase (MAPK) pathway, regulating growth and function in both normal and neoplastic cells. Several tumor models support the evidence that most of the DTCs may be due to single activating somatic mutations in one of three genes: *BRAF*, *RAS* and traslocations producing *RET/PTC* oncogenes [5]. Molecular signaling pathways are differently affected in different types of thyroid cancer [6]. For example, *BRAF* mutation is found preferentially in PTC conventional variant. The substitution of a thymidine to adenine in exon 15 results in an amino acid sequence change of valine to glutamate (V600E); this alteration makes *BRAF* constitutively active [16,17,18] with a higher affinity for MEK1 and MEK2 and with a potent activating effect on MAPK pathway. This mutation (V600E) does not seem related to radiation exposure, but some authors suggest that PTCs with *BRAF* mutations are associated with a more aggressive clinical pattern, advanced stage and extrathyroidal invasion [19]. Furthermore, *BRAF* mutation is common in aggressive microcarcinomas [20]; it is also correlated to a decreased expression of sodium-iodide symporter and so to the refractoriness to radioiodine therapy [21].

In DeDTC, *RET/PTC* rearrangements are found in 30–40%, *RAS* mutations in about 10%, and *BRAF* mutations in approximately 40–50%, with no overlap among these mutations in PTC, whereas a higher prevalence of *BRAF* mutations (up to 70%) has been observed in dedifferentiated papillary thyroid carcinoma (DePTC) [5,22,23]. *RET/PTC* rearrangements are correlated to radiation exposure and are found in pediatric PTC [24]. Twelve variants of *RET/PTC* have been decribed [25]. More recently, it has been stressed the relevance of alterations in the PI3K/AKT pathway in the mechanism of tumorigenesis and of dedifferentiation, particularly in follicular variant cancer [26]. Alterations in this signaling pathway were found in 31% of benign thyroid adenoma, 24% of PTCs, 55% of FTCs and 58% of anaplastic thyroid cancer (ATC), underlining its important role in the progression of benign lesion toward ATC [27].

## NEW AGENTS FOR THE TREATMENT OF THYROID CANCER: TKIS

Since 2005, a wide variety of multitargeted kinase inhibitors have entered clinical trials for patients with advanced or progressing metastatic thyroid cancers yielding higher response rates than cytotoxic chemotherapy, also if responses have been observed in only few patients [28]. Most of these agents have a common property of inhibiting vascular endothelial growth factor receptors (VEGFR), with a potent antiangiogenetic role, because of the structural similarity between RET and VEGFR kinases; *i.e.* Sorafenib has *RAF-RET*, and VEGFR-inhibiting activity; axitinib has VEGFR-, C-KIT-, and platelet-derived growth factor receptors (PDGFR)-inhibiting activity; pazopanib is an inhibitor of VEGFR and PDGFR; and sunitinib inhibits E7080 (a multi-kinase inhibitor) and VEGFR.

The toxicities (adverse effects) of these drugs differ slightly from the toxicities of mainstream anticancer drugs and they primarily consist of fatigue, hypertension, appetite loss, diarrhea, hypothyroidism and skin disease. Cases of hypothyroidism and severe cardiac impairment during TKIs therapy have been also described [29]. Because of the targeting similarities of many of TKIs, common toxicities exist among these agents [30].

Since these drugs are basically administered long-term, their anti-tumor effect decreases greatly when the dose must be reduced or administration must be discontinued because of such adverse effects.

The effects of these new therapies seem to be more potent in patients with PTC than with FTC or poorly differentiated carcinoma, in those with the mutated *BRAF* gene than with wild-type *BRAF*, and in those with lung rather than bone metastases [28].

Several phase II trials of different tirosine kinases inhibitor were conducted and others are ongoing (Table **1**). 

There have been three different phase II trials of sorafenib [BAY 43-9006, 400 mg bis in die (b.i.d.)]. The first trial of sorafenib (400 mg b.i.d.) was conducted on 30 DTC patients, and a partial response was reported in 7 patients and stable disease in 16 patients [31]. The second trial reported a partial response in 6 patients and stable disease for 6 months or more in 23 of the 41 patients with PTC, but therapy was ineffective in the 11 patients with FTC or poorly differentiated DTC and in the 4 patients with ATC [32]. In the third trial, which was conducted on 32 DTC patients, a partial response was reported in 8 patients and stable disease in 11 patients [33]. Greater efficacy of sorafenib was seen in PTC especially PTC with a *BRAF* mutation, than in poorly differentiated DTC, and it had no effect on iodine uptake. A phase III trial comparing progression-free survival is under way in which sorafenib and placebo are being administered to patients with radioiodine resistant metastatic DTC. 

On the other hand, several phase II trials of sunitinib (SU11248) are in progress and, according to the reports, thus far partial responses and stable diseases have been seen in DTC and MTC [34]. Carr *et al.* conducted a phase II study of continuous dosing of sunitinib in 35 patients affected by iodine-refractory DTC and MTC. Thirty three patients were evaluated for disease response according to RECIST: 1 complete response, 10 partial response and 16 stable disease patients were observed [35]. Progressive disease was seen in 6 patients [35].

Cohen *et al.* tested axitinib (starting dose, 5 mg twice daily) in sixty patients with advanced thyroid cancer of any histology. Responses were noted in all histologic subtypes. A partial response was observed in 18 patients and stable disease in another 23 patients [36].

Recently, Verbeek *et al.* [37] compared the effect of four TKIs (axitinib, sunitinib, vandetanib and XL184) on cell proliferation, RET expression and autophosphorylation and extracellular signal-regulated kinases (ERK) activation in cell lines expressing a MEN2A, MEN2B mutations and a *RET/PTC* rearrangement, showing a most potent effect of XL184 (an inhibitor of VEGF receptors 1 and 2, C-MET, RET, C-KIT, FLT3, and Tie-2 [38]) in MEN2A and PTC and a more effective action of vandetanib in MEN2B *in vitro*. 

The TKI imatinib (STI571) was tested in patients with metastatic MTC yelding no objective responses but stable disease in a minority of patients [39], while motesanib, in a mouse model of MTC, inhibited tumor xenograft growth reducing directly VEGFR 2 and RET expression [40]. 

A phase II study assessed the efficacy of Vandetanib (300 mg/day), a selective inhibitor of VEGFR2, epidermal growth factor receptor (EGFR) and RET, in patients with advanced hereditary MTC. Thirty patients were enrolled: 20% of patients had a partial response, while an addictional 53% of patients experienced a stable disease at 24 weeks. A reduction in calcitonin and carcinoembryonic antigen levels was also reported [41]. Another study evaluated vandetanib in 19 patients with advanced hereditary MTC, yielding a partial response in 3 patients, stable disease lasting 24 weeks or longer in further 10 patients with decreasing levels of calcitonin and carcinoembryonic antigen levels (a sustained 50% or greater decrease from baseline in 3 of 19 patients and 5% in 1 of 19 patients, respectively) [42]. 

Pazopanib (800 mg daily in 4-week cycles), an inhibitor of VEGFR and PDGFR, was tested in patients with metastatic, rapidly progressive and radioiodine refractory DTC and it seems to represent a promising therapeutic option (confirmed partial response in 18 patients of the 39 enrolled) [43].

A phase II trial of gefitinib (250 mg/day) has been performed in 27 patients [subtypes of thyroid cancer included: papillary (n=11), follicular (n=6), anaplastic (n=5), medullary (n=4), and Hurthle cell carcinomas (n=1)], but no antitumor effect was observed [44]. Gefitinib and vandetanib were tested in three thyroid cancer cell lines (ATC, FTC and PTC) and resulted as a potent inhibitors of EGF-R and VEGF-R with consequently reduction in cell proliferation and VEGF secretion [45].

In a metastatic murine model of MTC, it has been recently tested a novel RET inhibitor, withaferin A, that was responsible for tumor regression and growth delay [46]. 

## TARGETED THERAPIES LIMITS AND SIDE EFFECTS

Tumor cells often devise strategies to bypass the effects of antineoplastic agents and selection of therapy-resistant clones is frequently the reason for treatment failure.

Imatinib treated chronic myeloid leukemia patients relapse is due to accumulation of very specific point mutations within the catalytic domain of the ABL kinase [47]. 

A lack of response can occur, for instance, because target inhibition raises the activity of compensatory signal pathways, which, in turn, rescue tumor cell growth. However, the possibility of testing the sensitivity of primary DePTC cells from each subject to different TKIs could increase the effectiveness of the treatment. In fact, *in vitro* chemosensitivity tests are able to predict *in vivo* effectiveness in 60% of cases [48]; while, it is well known that a negative chemosensitivity test *in vitro* is associated with a 90% of ineffectiveness of the treatment *in vivo* [47], allowing the administration of inactive chemotherapeutics to these patients to be avoided [48]. It has been recently demonstrated that it is possible to test the antineoplastic activity of different compounds [antiblastics or peroxisome proliferator-or-activated receptor (PPAR)-γ agonists] in primary anaplastic thyroid cancer cells obtained from each patient [49, 50]. Furthermore, it has been shown that primary cells can be obtained directly from fine needle aspiration samples of thyroid cancer, and that the results of *in vitro* chemosensitivity tests are quite similar to those obtained from surgical biopsies [51]. Interestingly, more recently, we have first shown an antitumoral effect of two new multi-targeted kinases inhibitors (CLM3 and CLM29) not on continuous cell lines, that are quite different from the tumor of the patients themselves, but directly on primary DePTC of patients refractory to the radioiodine therapy [52], opening the way to the possibility of personalizing the kinase inhibitors therapy in each patient. 

Numerous side effects of the multi-targeted kinases inhibitors were reported; in different trials, several patients required a dose reduction to improve tolerability. The most common side effects involved cardiovascular system (hypertension, cardiomyopathy, stroke) and skin (rashes, foot and hand syndrome) [28]. Some cases of TSH elevation were also observed, especially during motesanib therapy [53]. 

## CONCLUSION

The most encouraging results, in patients with advanced DTC and DeDTC unresponsive to radioiodine therapy and MTC, were obtained with the targeted kinase inhibitors with an intrinsic activity against VEGFR and cross activity against RET kinases, such as pazopanib, sorafenib, motesanib, sunitib and vandetanib. Also axitinib, a more specific inhibitor of VEGFR, showed a great efficacy [36]. Unfortunately, eventual progression despite antiangiogenic VEGFR blockade suggests emergence of alternate pathways to promote tumor growth and metastasis (including FGFR, C-MET, and angiopoietins) [54].

Until now, no consensus guidelines or standard criteria for patients enrolling were established definitively. Thereby the effects on survival are unclear, because of lack of complete responses and because of the discrepancies between the radiographic tumor responses and the effective improvement of survival [30].

The aim of the introduction of these targeted therapies is to extend life duration assuring a good quality of life. To reach these goals, we need to have further data on toxicities of single agent and of combination of more drugs, to identify specific biomarkers able to predict the treatment efficacy, the clinical outcome and to guarantee a tailored dosage of the drugs. Moreover, the possibility to test *in vitro* (in primary thyroid cancer cells) these novel drugs may help to improve further the personalization of the treatment. 

## Figures and Tables

**Table 1.** Clinical Trials with Targeted Therapies in Patients with Thyroid Cancer

Author and year of the study	Drug	Pathway inhibited	Thyroid cancer	Responses

Gupta-Abramson *et al*. 2008	Sorafenib	RAF, RET, VEGFR	30 DTC	7 (23%)PR;
				16 (53%)
				SD (DTC)

Kloos *et al*. 2009	Sorafenib	RAF, RET, VEGFR	41 PTC	6 (15%)PR;
			11 FTC	23 (56%) SD (PTC)
			4 ATC	No response (FTC)
				No response (ATC)

Hoftijzer *et al*. 2009	Sorafenib	RAF, RET, VEGFR	32 DTC	8 (25%)PR;
				11 (34%)SD (DTC)

Cohen *et al*. 2008	Sunitinib	E7080, VEGFR	37 DTC	4 (11%)PR; 21 (57%)SD
			6 MTC	(DTC)
				2 (33%)SD (MTC)

Carr *et al*. 2010	Sunitinib	E7080, VEGFR	28 DTC	1 (3%)CR
			7 MTC	10 (29%)PR
				16 (46%)SD

Cohen *et al*. 2008	Axitinib	VEGFR, C-KIT, PDGFR	60 pts of any histology	18 (30%)PR, 23 (28%)SD

de Groot 2007	Imatinib	VEGFR 2, RET	15 MTC	4 (27%)SD (MTC)

Wells *et al*. 2010	Vandetanib	VEGFR 2, EGFR, RET	30 MTC hereditary	6 (20%)PR,
				16 (53%)SD (MTC)

Robinson *et al*. 2010	Vandetanib	VEGFR 2, EGFR, RET	19 MTC hereditary	3 (16%)PR,
				10 (53%)SD (MTC)

Bible *et al*. 2010	Pazopanib	VEGFR, PDGFR	39 DeDTC	18 (46%) PR (DeDTC)

Pennel *et al*. 2008	Gefitinib	EGFR	27 patients of different tumor types	No response

VEGFR = vascular endothelial growth factor receptor; PDGFR = platelet-derived growth factor receptors; EGFR = epidermal growth factor receptor. DTC = differentiated thyroid cancer; ATC = anaplastic thyroid cancer; PTC = papillary thyroid cancer; FTC = follicular thyroid cancer; DeDTC = dedifferentiated differentiated thyroid cancer; MTC = medullary thyroid carcinoma. PR = partial response; SD = stable disease; CR = complete response.

## ABBREVIATIONS

ATC: = Anaplastic thyroid cancer
b.i.d.: = Bis in die
CR: = Complete response
DeDTC: = Dedifferentiated differentiated thyroid cancer
DePTC: = Dedifferentiated papillary thyroid carcinoma
DTC: = Differentiated thyroid cancer
EGFR: = Epidermal growth factor receptor
ERK: = Extracellular signal-regulated kinases
FMTC: = Familial medullary thyroid carcinoma
FTC: = Follicular thyroid cancer
MAPK: = Mitogen activated protein kinase
MEN2: = Multiple Endocrine Neoplasia Type 2
MTC: = Medullary thyroid carcinoma
PDGFR: = Platelet-derived growth factor receptors
(PPAR)-γ: = Peroxisome proliferator-activated receptor-γ
PR: = Partial response
PTC: = Papillary thyroid cancer
SD: = Stable disease
TKIs: = Tyrosine kinase inhibitors
TSH: = Thyroid-stimulating hormone
VEGFR: = Vascular endothelial growth factor receptor
